# Intrapulmonary solitary fibrous tumour: a case report

**DOI:** 10.1093/jscr/rjaa603

**Published:** 2021-02-18

**Authors:** Akie Horikiri, Hiroyoshi Tsubochi, Natsuki Mizukoshi, Ryota Myobatake, Hidetaka Sakurai, Tomoki Shibano, Yoshihiko Kanai, Shinichi Yamamoto

**Affiliations:** Department of General Thoracic Surgery, Jichi Medical University, Tochigi Japan; Department of General Thoracic Surgery, Jichi Medical University, Tochigi Japan; Department of General Thoracic Surgery, Jichi Medical University, Tochigi Japan; Department of General Thoracic Surgery, Jichi Medical University, Tochigi Japan; Department of General Thoracic Surgery, Jichi Medical University, Tochigi Japan; Department of General Thoracic Surgery, Jichi Medical University, Tochigi Japan; Department of General Thoracic Surgery, Jichi Medical University, Tochigi Japan; Department of General Thoracic Surgery, Jichi Medical University, Tochigi Japan

## Abstract

Solitary fibrous tumours (SFTs) mainly originate from the visceral pleura and may protrude to the thoracic cavity, but intrapulmonary SFTs are extremely rare. We describe a rare case of SFT arising in the right lung of an 83-year-old man who underwent surgical excision. Chest computed tomography (CT) revealed a 10-mm tumour in the lower lobe of the right lung. The size of tumour gradually increased and reached 17 mm 2 years after the first radiologic examination. Considering the possibility of malignancy, wedge resection of the right lower lobe was performed via video-assisted thoracic surgery. Microscopically, the tumour consisted mainly of spindle-shaped cells. Immunohistochemical staining indicated the tumour was positive for CD34, STAT6, vimentin and bcl-2, but negative for cytokeratins, D2–40 and S-100. Based on the histological findings, the tumour was diagnosed as SFT. The patient has been in good health for 6 months since the surgery.

## INTRODUCTION

Solitary fibrous tumours (SFTs) usually arise in the visceral pleura and less frequently in a wide variety of extra-pleural locations. Although SFTs can occur in the pulmonary parenchyma [1], intrapulmonary SFTs are rare. Preoperative diagnosis of intrapulmonary SFT is difficult due to its rarity and non-specific radiological findings. Here, we describe a case of intrapulmonary SFT that slowly grew during a 2-year follow-up computed tomography (CT).

**Figure 1:**
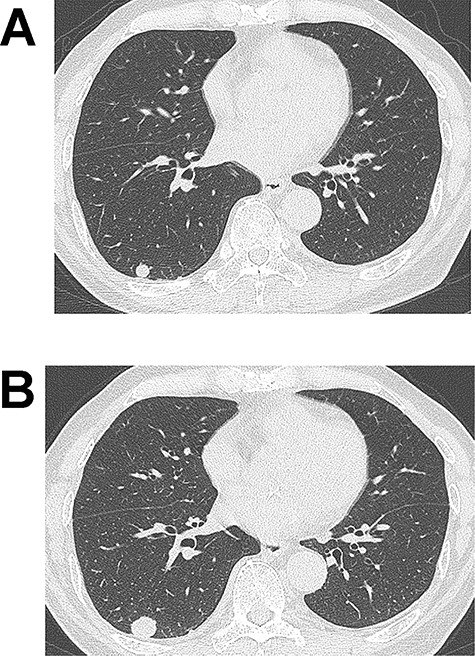
**(A)** Computed tomography (CT) image showing a 10-mm nodule in the lower lobe of the right lung. (**B)** The tumour grew to 17 mm in diameter on CT 2 years after the initial imaging.

## CASE REPORT

An 83-year-old man with a 60-pack-year history of smoking was found to have an abnormal shadow on chest radiography. He was asymptomatic and laboratory test results, including tumour markers, were all normal. Chest CT revealed a 10-mm-diameter tumour in the lower lobe of the right lung (Fig. 1A). Since the tumour was well demarcated, it was suspected to be a benign lesion such as hamartoma, and the patient underwent a conservative follow-up approach. The tumour size gradually increased on CT during follow-up and subsequently reached 17 mm 2 years after the initial imaging (Fig. 1B). At that time, ^18^F-fluoro-deoxy-glucose positron emission tomography (FDG-PET) showed slight uptake in the right lung tumour (maximum standardized uptake: 1.71). Since malignancy could not be completely ruled out, surgery was scheduled. Wedge resection of the right lower lobe was performed using video-assisted thoracic surgery. Intraoperative frozen section diagnosis revealed a tumour with spindle cells that did not contain a malignant component.

**Figure 2:**
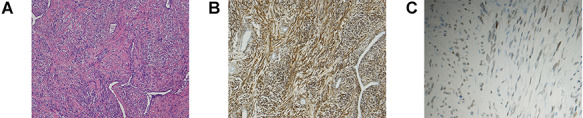
**(A)** H&E staining of the resected tumour revealed proliferating spindle-shaped cells with a random fascicular arrangement and the deposition of wavy hyalinized collagen. (**B, C)** Immunohistochemical staining of the tumour. The tumour cells were positive for CD34 (B). Nuclear positivity for STAT6 was observed (C).

Macroscopically, the tumour was a well-circumscribed, yellowish-white mass measuring 17 mm in diameter. Microscopically, the tumour showed mainly spindle-shaped cells (Fig. 2A) and did not involve the pleura. Neither intra-tumoural necrosis nor bleeding was observed. Mitotic figures were observed at 0–1 per 10 high-power fields (×400). The background was fibrous tissue in a patternless arrangement. Immunohistochemical staining indicated that the tumour was positive for CD34 (Fig. 2B), STAT6 (Fig. 2C), vimentin and bcl-2 but negative for cytokeratins, D2–40 and S-100. The Ki-67 labelling index of the tumour was 10–20%. Based on these histological findings, the tumour was diagnosed as SFT. The patient has been in good health for 6 months after the surgery without recurrence.

## DISCUSSION

SFTs arising from the visceral pleura may rarely grow inward into the peripheral lung [2]. However, the tumour in the present case was not compatible with such an SFT as it did not involve the visceral pleura. We speculate that this tumour arose from the lung parenchyma. Two main explanations for intrapulmonary SFTs have been proposed [3]. First, the subpleural mesenchyme is in direct continuity with the connective tissues of the intralobular septa, and intrapulmonary fibrosis may arise from this mesenchyme. Second, the tumours may originate from fibroblasts in the pulmonary parenchyma.

Several previous reports have included radiological findings of intrapulmonary SFTs and described them as well-demarcated tumours [4–6]. Benign SFTs show heterogeneously low FDG uptake in FDG-PET, as in our case [5, 6], and high FDG uptake suggests malignant SFTs [4]. There are little data on serial changes on CT. In our case and that in another report [6], a small, well-circumscribed nodule on CT slowly grew during follow-up.

Immunohistochemical examination is useful for differentiating SFTs from other neoplasms. Although CD34 immunoreactivity has traditionally been used to differentially diagnose SFTs, it is not specific. Nuclear staining of STAT6 has been more recently established as a highly sensitive and specific marker of SFTs and is currently widely used for differential diagnosis of SFTs. STAT6 staining is also useful to reveal from other neoplasms revealing spindle cell proliferation [7, 8].

Although SFTs are usually slow-growing and have a favourable prognosis, some are malignant and eventually result in death because of local recurrence or metastatic disease [4]. The histological criteria for malignant SFTs include high cellularity, high mitotic activity (defined as more than four mitotic figures per 10 high-power fields), pleomorphism, and necrosis or haemorrhage [2]. Immunohistochemically, Ki-67 has been reported to be a useful marker for distinguishing malignant SFTs from benign SFTs. The Ki-67 labelling index has been reported to vary from 1 to 44% in malignant tumours and from 0 to 2% in benign tumours [9]. In the present case, the tumour was determined to be benign because it did not match the histological criteria for malignant SFTs based on H&E staining. However, careful follow-up is necessary because the Ki-67 labelling index was 10–20%.

In conclusion, we experienced an extremely rare case of intrapulmonary SFT that was resected after 2 years of follow-up on CT. Although preoperative diagnosis of SFT is difficult, we should be aware that slow-growing, well-circumscribed pulmonary nodules may be anSFT.

## CONFLICT OF INTEREST STATEMENT

None declared.

## FUNDING

None.
